# Immune Cell Molecular Pharmacodynamics of Lanreotide in Relation to Treatment Response in Patients with Gastroenteropancreatic Neuroendocrine Tumors

**DOI:** 10.3390/cancers16173104

**Published:** 2024-09-07

**Authors:** Sabah Alaklabi, Orla Maguire, Harsha Pattnaik, Yali Zhang, Jacky Chow, Jianmin Wang, Hans Minderman, Renuka Iyer

**Affiliations:** 1 Department of Medicine, Roswell Park Comprehensive Cancer Center, Buffalo, NY 14263, USA; sfalaklabi@kfshrc.edu.sa (S.A.);; 2 Flow & Image Cytometry Shared Resource, Roswell Park Comprehensive Cancer Center, Buffalo, NY 14263, USA; orla.maguire@roswellpark.org (O.M.);; 3 Department of Biostatistics & Bioinformatics, Roswell Park Comprehensive Cancer Center, Buffalo, NY 14263, USA; 4 Department of Immunology, Roswell Park Comprehensive Cancer Center, Buffalo, NY 14263, USA

**Keywords:** lanreotide, neuroendocrine tumors, immune response, somatostatin analogs

## Abstract

**Simple Summary:**

Somatostatin analogs like lanreotide are considered first-line agents for the treatment of gastroenteropancreatic neuroendocrine tumors (NET). Although its effects on tumor proliferation and hormonal regulation are somewhat understood, its influence on the immune system has not been well elucidated. We used a double-pronged approach to understand the role of lanreotide in immune system regulation in healthy donor T cells in vitro as well as in vivo in cells obtained from 17 NET patients. We looked at cytokine signaling and differential gene expression to elucidate lanreotide’s role in the treatment of NET through additional novel immune pathways.

**Abstract:**

The CLARINET trial led to the approval of lanreotide for the treatment of patients with gastroenteropancreatic neuroendocrine tumors (NETs). It is hypothesized that lanreotide regulates proliferation, hormone synthesis, and other cellular functions via binding to somatostatin receptors (SSTR1–5) present in NETs. However, our knowledge of how lanreotide affects the immune system is limited. In vitro studies have investigated functional immune response parameters with lanreotide treatment in healthy donor T cell subsets, encompassing the breadth of SSTR expression, apoptosis induction, cytokine production, and activity of transcription factor signaling pathways. In our study, we characterized in vitro immune mechanisms in healthy donor T cells in response to lanreotide. We also studied the in vivo effects by looking at differential gene expression pre- and post-lanreotide therapy in patients with NET. Immune-focused gene and protein expression profiling was performed on peripheral blood samples from 17 NET patients and correlated with clinical response. In vivo, lanreotide therapy showed reduced effects on wnt, T cell receptor (TCR), and nuclear factor kappa-light-chain-enhancer of activated B cells (NF-kB) signaling in CD8+ T cells in responders compared to non-responders. Compared to non-responders, responders showed reduced effects on cytokine and chemokine signaling but greater effects on ubiquitination and proteasome degradation genes. Our results suggest significant lanreotide pharmacodynamic effects on immune function in vivo, which correlate with responses in NET patients. This is not evident from experimental in vitro settings.

## 1. Introduction

Our understanding of neuroendocrine tumors (NETs) has improved over the last decade. However, data from the Surveillance, Epidemiology, and End Results (SEER) database show that overall survival for stage IV NETs remains dismal, highlighting the need for further research to improve on current therapies [1]. Radioligand and targeted therapies have recently demonstrated efficacy in randomized clinical trials and have been approved, but somatostatin analogs (SSAs) are still considered the backbone of the treatment of NETs [2,3,4]. Due to SSA-induced gall bladder hypomotility, careful periodic follow-up must be performed for NET patients to look for cholelithiasis and associated complications [5]. Lanreotide autogel/depot as a long-acting SSA binds with high affinity to type 2 somatostatin receptor (SSTR2) and SSTR5 [6,7], suggestive of a role in the inhibitory effect of lanreotide on tumor cell proliferation and hormone secretion. The SSTRs are highly expressed on NETs, and a higher degree of receptor expression is correlated with the degree of differentiation [8,9]. Hence, SSAs are effective only in grade 1 and 2 NETs and not in neuroendocrine cancers (NECs). The landmark CLARINET trial studied the effect of lanreotide 120 mg in patients with untreated metastatic or locally advanced, well-differentiated, and non-functioning pancreatic and intestinal NETs that expressed SSTR by nuclear imaging. Progression-free survival (PFS) was shown to be significantly increased in the treatment group, with long-term data confirming the benefit of PFS, which was observed regardless of tumor burden [10,11,12].

It has been demonstrated in multiple studies that immune cells express SSTR1–5 differentially, and a growing body of evidence indicates that somatostatin hormone may influence the immune system and the regulation of T cell functions [13,14,15,16,17,18,19]. Despite these findings, the exact effect of SSAs on T cells and their function is not understood. Immunotherapy has shown remarkable improvements in the survival of patients with various solid tumors like melanoma and non-small cell lung cancer [20,21]. This heralds interest in investigating the immunotherapeutic effects of lanreotide in NET and subsequently developing pharmacotherapy that works on similar pathways. Preclinical studies to gain insight into the effect of SSAs on the immune system are needed to provide rationale and establish a foundation for clinical trials investigating the role of combination treatments with immunotherapies. Moreover, it may aid in identifying predictive biomarkers for SSA responses that are needed to facilitate the selection of patients who may benefit. 

We conducted this study to investigate the effects of lanreotide on peripheral blood T cells in vitro and in vivo and to validate them in patient samples using clinical response correlates. Additionally, we sought to evaluate key functional parameters of the immune response in these cells.

## 2. Materials and Methods

### 2.1. In Vitro Assay

#### 2.1.1. Specimens 

Peripheral blood samples were collected from healthy donors and NET patients according to protocols approved by the Institutional Review Board (IRB) at Roswell Park Comprehensive Cancer Center (IRB 03103 and 116019). Written informed consent for participation was obtained in accordance with national legislation and institutional requirements. The study was conducted in compliance with the Declaration of Helsinki and Good Clinical Practice Guidelines. 

#### 2.1.2. SSTR Expression in Peripheral Blood 

Healthy donor peripheral blood samples were phenotyped for the T cell surface markers CD3, CD4, CD8, CD25, and CD127, following which red blood cells (RBCs) were lysed, white blood cells (WBCs) were fixed and permeabilized, and cells were stained with one of the 5 SSTR antibodies and a fluorescent secondary antibody. (Appendix A Table A1) Samples were acquired using an LSRFortessa (Becton Dickinson, Franklin Lakes, NJ, USA) and analyzed using WinList v8.0 (Verity Software House, Topsham, ME, USA). SSTR levels were expressed as the relative increase in mean fluorescence intensity (MFI) of staining with the primary unconjugated antibody in combination with the fluorescently conjugated secondary antibody compared to the MFI of staining with the fluorescently conjugated secondary antibody only. Full antibody details are included in Appendix A Table A1.

#### 2.1.3. Intracellular IL-2 and IFNg Expression 

Healthy donor peripheral blood was pre-treated with lanreotide (Selleck chemicals, Houston, TX, USA) at concentrations up to 10 mcM overnight at 37 °C/5% CO_2_ before incubation with or without Phorbol-12-myristate-13-acetate (PMA)/Ionomycin/Brefeldin A mix for 4 h at 37 °C/5% CO_2_. For flow cytometry, the cells were labeled with the surface markers CD3, CD4, CD8, CD25, and CD127. RBCs were then lysed, and WBCs were fixed and permeabilized. The cells were then stained with Interleukin-2 (IL-2) and Interferon gamma (IFNg), acquired, and analyzed as above. 

#### 2.1.4. Apoptosis Assay 

Healthy donor peripheral blood was treated with PMA/Ionomycin overnight at 37 °C/5% CO_2_ with or without lanreotide at concentrations up to 10 mcM. RBCs were then lysed, and apoptosis in WBCs was measured using CellEvent Caspase-3/7 Green reagent (ThermoFisher, Cat # C10423) according to manufacturers’ instructions. Cells were acquired and analyzed by flow cytometry, as shown above.

#### 2.1.5. Transcription Factor Assays 

Healthy donor peripheral blood was pre-treated with lanreotide at concentrations up to 5 mcM overnight at 37 °C/5% CO_2_ before incubation with or without PMA/Ionomycin for 0.5 h at 37 °C/5% CO_2_ or anti-CD3/CD28 antibodies for 2 h at 37 °C/5% CO_2_. Cell surface phenotyping was performed as described above for SSTR expression. RBCs were then lysed, WBCs were fixed and permeabilized, and cells were stained with one of the two nuclear factors of activated T-cells (NFAT1)—nuclear factor kappa-light-chain-enhancer of activated B cells (NF-kB p65) or extracellular signal-regulated kinase (ERK1/2). Cells were then stained with a secondary donkey anti-rabbit AlexaFluor647 antibody. Samples were acquired using an ImageStreamX Mk-II (Amnis, part of Cytek Biosciences, WA, USA) and analyzed using IDEAS v6.2 as previously described [22]. In brief, the nuclear translocation of the transcription factor of interest is quantified on an individual cell level in immunophenotypically defined cell populations using a so-called similarity score. The similarity score is a parameter that quantifies the degree of similarity between a nuclear stain image and a corresponding transcription factor-specific stain image of the same cell. The greater the similarity score (SS), the more nuclear the localization of the transcription factor is.

### 2.2. In Vivo Analyses

#### 2.2.1. Patient Selection Criteria

The following criteria were required for patients’ selection: newly diagnosed, histologically confirmed unresectable, measurable, locally advanced, or metastatic, well, or moderately differentiated (according to WHO 2017 classification) functionally inactive NET of any primary site. Consent must have been provided to obtain peripheral blood mononuclear cells at baseline, before starting lanreotide, and 3 months after starting lanreotide. Availability of follow-up data for clinical response and survival was required. Clinical response data were collected by the Roswell Park Comprehensive Cancer Center’s Biomedical Research Informatics Shared Resource.

#### 2.2.2. Treatment Response

The definition of response adopted in this study is a combination of clinical improvement and no progression on CT scans by 19 months. Response assessment in NETs is not commonly defined solely based on radiographic improvement since SSAs primarily cause tumor stability rather than shrinkage, and monitoring changes in tumor size for response assessment is considered inadequate [23]. A patient was labeled as a non-responder if the treating physician felt the need to switch to or add the next line of therapy.

#### 2.2.3. Treatment Schedule

Lanreotide autogel/depot (Ipsen Pharmaceuticals) was administered in 120-mg deep subcutaneous injection every 28 days. No other anticancer medications were allowed during the study.

#### 2.2.4. Sorting of T Cell Subpopulations for NanoString Analysis

**Figure A1:**
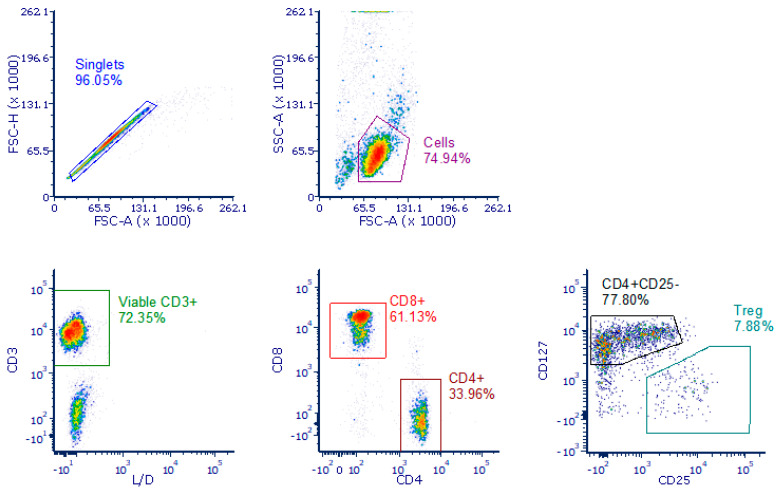
Gating strategy used in sorting T cell sub-populations. Isolating T cells for sorting followed a hierarchical gating strategy where, from left to right, upper to lower, singlets were selected based on forward scatter area against height. A lymphoid population was then selected based on forward scatter area against side scatter area. (Cells). Viable T cells were selected as CD3 positive and negative for LiveDead viability dye. CD4 negative, CD8 positive TC cells were selected. CD8 negative, CD4 positive were further gated as either CD25 negative, CD127 positive TH cells or CD127 dim/negative, CD25 positive Treg cells.

To determine the in vivo relationship between expression/transcription on relevant immune response proteins in T cells and response to lanreotide, viably procured peripheral blood mononuclear cells from NET patients were analyzed by a Counter Vantage 3D™ RNA:Protein Immune Cell Profiling Assay (NanoString, Inc. Seattle, WA, USA). This assay integrates immune cell flow sorting with downstream multiplexed detection of 770 RNA and 30 cell surface proteins from the flow-sorted specimen. Frozen peripheral blood mononuclear cells from NET patients were thawed and incubated with oligo-tagged NanoString antibodies (part of the Vantage 3D RNA:Protein Immune Cell profiling Assay kit) in addition to the NanoString compatible fluorescent antibodies CD3, CD8, CD4, CD25, and CD127, alongside LiveDead (LD) fixable violet viability dye (ThermoFisher, Waltham, MA, USA). Three populations were sorted into Buffer RLT (ThermoFisher) using an Aria II cell sorter (Becton Dickinson). Populations were 1. LD-CD3+CD4-CD8+ (cytotoxic T cells); 2. LD-CD3+CD8-CD4+CD25-CD127+ (T helper cells); 3. LD-CD3+CD8-CD4+CD25+CD127-(T regulatory cells). An example of the gating strategy is shown in Appendix A Figure A1. Samples were stored at −80 °C until NanoString assay.

#### 2.2.5. NanoString Assay

Two cell lysates from each previously flow-sorted T cell subpopulation were produced for nCounter hybridization and processing. A denatured cell lysate for protein hybridization was combined with the remaining cell lysate for RNA hybridization to Nanostring reporter and capture tagsets, followed by post-hybridization processing by the nCounter Prep station. Data collection was carried out in the nCounter Digital Analyzer, where digital images are processed, and the barcode counts are tabulated in a comma-separated value (CSV) format. The normalized data generated with the Nanostring nSolver software (version 4.0.70) were used for data analysis.

#### 2.2.6. Statistical Analysis of NanoString Data

Paired samples obtained before and 3 months into treatment were analyzed for each patient. For the analysis, a linear mixed model (LMM) was fitted to test three different effects. The ‘patient effect’ analyses were performed to evaluate if any gene was significantly different between responders and non-responders at the pre-treatment time point. This analysis tests the predictive value of observed pre-treatment differences on treatment outcome, where non-responders served as reference. The positive and negative *t*-values reflect higher or lower gene expression levels, respectively, in responders relative to non-responders.

The ‘Treatment effect’ estimated if any genes were significantly changed before and after treatment (regardless of response). For this analysis, the pre-treatment samples served as a reference. The positive and negative *t*-values reflect higher or lower gene expression levels, respectively, after treatment relative to pre-treatment (regardless of outcome).

The ‘Interaction Effect’ estimated if the degree of difference in gene expression before and after treatment in responders versus non-responders was significant. For example, a gene’s expression between responder and non-responder pre-treatment may be similar, but it may change significantly at post-treatment, and vice versa. In both cases, the non-responders served as the reference. The positive and negative *t*-values are associated with ‘greater’ or ‘lesser’ effects, respectively, observed in responders relative to non-responders. If the treatment had a larger effect on responders, the *t* value will be positive regardless of whether the gene was significantly up- or downregulated in those responders. Benjamin–Hochberg multiple test adjustment was carried out, and the adjusted-*p* values were reported. The analysis was carried out using R statistical software (version 4.2). The significantly changed genes with adjusted *p*-value < 0.05 were used in a gene set enrichment analysis (GSEA) to identify enriched pathways with all genes on the panel used as background using gProfiler2 [24].

## 3. Results

### 3.1. In Vitro Results

#### 3.1.1. SSTR Expression on T Cells

**Figure 1:**
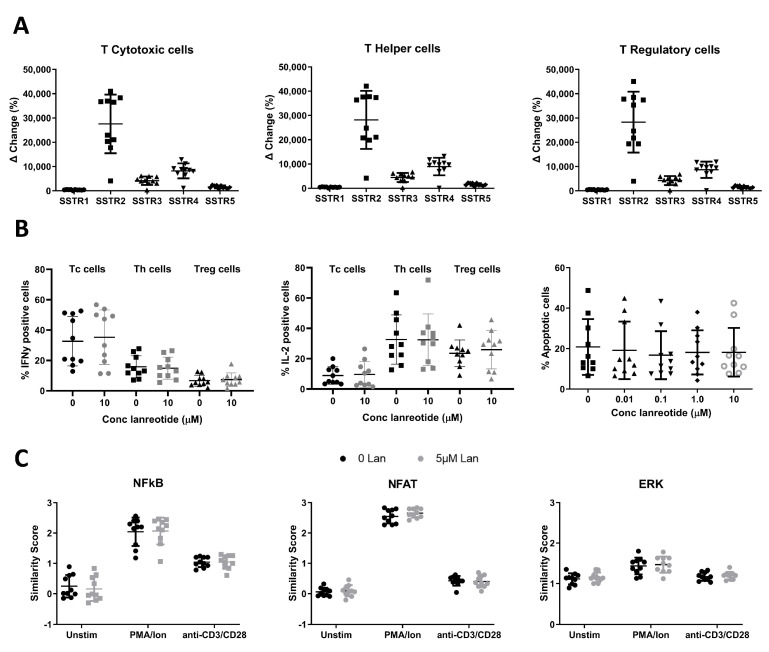
SSTR expression and in vitro effects of lanreotide on healthy human T cell function and survival. (**A**) Expression of SSTR receptors 1–5 on Tc (**left** graph), Th (**middle** graph), and Treg (**right** graph) cell populations showing SSTR2 to be the most abundantly expressed SSTR in all T cell populations studied. (**B**) Effects of in vitro lanreotide exposure on T cell function and survival. Function measured as IFNg (**left** graph) and IL-2 (**middle** graph) production, and survival measured by apoptosis assay (**right** graph). (**C**) Effects of in vitro lanreotide exposure on transcription factor signaling measured by activity of NFAT (**left** graph), NFkB (**middle** graph) or ERK1/2 (**right** graph). N = 10 for all experiments.

The expression of SSTR receptors 1–5 on T cells was assessed by flow cytometry on 10 healthy donor peripheral blood samples (Figure 1A). Cytotoxic T cells (Tc), helper T cells (Th), and regulatory T cells (Treg) were identified as CD3+CD4-CD8+ (Tc), CD3+CD8-/CD4+ (Th), and CD3+CD8-CD4+CD127dim/-CD25bright (Treg). The expression profile of the SSTRs was similar among the three T cell subsets, with a predominant expression of SSTR2. Compared to SSTR2, expression levels lowered by approximately 3-fold for SSTR4 and with diminishing expression levels of SSTR3 > SSTR5 and SSTR1, respectively.

#### 3.1.2. In Vitro Effects of Lanreotide on T Cell Function and Survival

The in vitro effect of lanreotide exposure on healthy donor T cell function was determined using 2 functional assays (Figure 1B). First, the effects on intracellular cytokine production (IL-2 or IFNg) were studied by flow cytometry following treatment with PMA/Ionomycin (PMA/Ion) in the absence and presence of lanreotide. On average, PMA/Ion-induced IFNg production was highest in Tc cells, 32.7 ± 5.1%, compared to 15.8 ± 2.3% and 6.7 ± 1.1% in Th and Tregs, respectively. PMA/Ion induced IL-2 production most effectively in Th cells with a mean positive population of 32.6 ± 5.1 (SE) % compared to 23.6 ± 2.8% and 8.9 ± 1.8% in Tregs and Tc cells, respectively. Lanreotide at concentrations up to 10 mcM had no significant effects on the PMA/Ion-induced IL-2 or IFNg production. For comparison, Figure 1B shows the data for both the untreated and the highest concentration of lanreotide tested (10 mcM). Results with the lower concentrations tested were not significantly different. The effect of the drug exposure on the overall survival of lymphocytes was determined by the induction of apoptosis (measured by the Caspase 3/7 activity with the cell-event assay) following exposure to PMA/Ion in the absence and presence of lanreotide. The data in Figure 1B (right plot) demonstrate that there were no statistically significant effects of the presence of the lanreotide doses tested on lymphocyte cell survival.

Next, the effect of lanreotide on key response signaling pathways was studied following treatment with PMA/Ion or anti-CD3/CD28 antibodies in the absence and presence of the drug by quantifying NFAT1, NF-kB, and ERK1/2 activity (Figure 1C).

The activation potential of the three pathways in the three T cell subsets studied was similar, with a more robust activation (relative similarity score (SS) increase from baseline values) observed following PMA/Ion exposure for the NFAT pathway compared to NF-kB and ERK1/2. Anti-CD3/CD28 antibodies more robustly activated NF-kB compared to the NFAT and ERK1/2 pathways. Lanreotide at concentrations up to 5 mcM had no significant effects on the PMA/Ion- or anti-CD3/CD28-induced activation of NFAT, NF-kB, or ERK1/2. Appendix A Table A2 summarizes the data for all three pathways as the mean similarity values obtained from 10 healthy donors with the associated standard errors (SE).

### 3.2. In Vivo Results

#### 3.2.1. Patient Characteristics

Between January 2019 and June 2021, 20 patients consented and met eligibility. Gene and protein expression profiling in sorted T cell subsets, performed using a NanoString immune cell panel, was limited to 17 patients. Of the three excluded patients, one died before the post-treatment sample, and two did not receive more than one lanreotide dose. Seven patients with intestinal NETs, nine with pancreatic, and one patient with an unknown primary site who were on treatment with lanreotide were analyzed.

The studied patient cohort consisted of nine responders (no progression by 19 months) and eight non-responders (Table 1). In both responders and non-responders, the liver was the most common metastasis site. Ki-67 score was generally lower in responders, and almost all patients had well-differentiated tumors.

#### 3.2.2. Proteomic and Transcriptional Analysis of T Cells of NET Patients in Relation to Lanreotide Response

**Figure 2:**
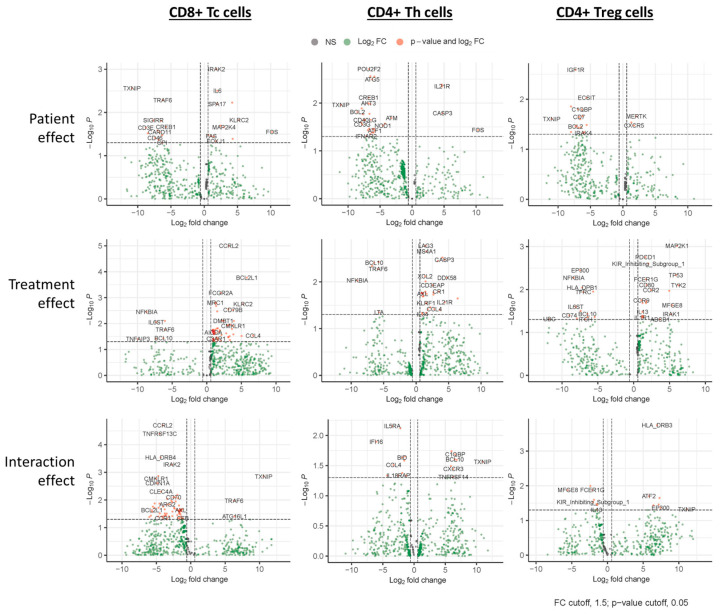
Proteomic and transcriptional analysis of in vivo lanreotide treatment in T cell subsets from NET patients. Paired samples obtained before and after treatment were analyzed for each patient. Volcano plots show a linear mixed model (LMM) analysis of Tc (**left** graphs), Th (**middle** graphs), and Treg (**right** graphs) cell populations fitted to test three different effects. The ‘Patient Effect’ (**upper** graphs) estimated if any gene was significantly different between Responder and Non-responder pre-treatment, where non-responders served as reference. The ‘Treatment effect’ (**middle** graphs) estimated if any genes were significantly changed between pre-treatment and post-treatment (regardless of response). For this analysis, the pre-treatment samples served as a reference. The ‘Interaction Effect’ estimated whether any genes were significantly changed before and after treatment in responders versus non-responders, where non-responders served as reference (lower graphs). Plots show the statistical significance (*p* value) versus the magnitude of change (fold change) for each of the 9 categories.

**Figure A2:**
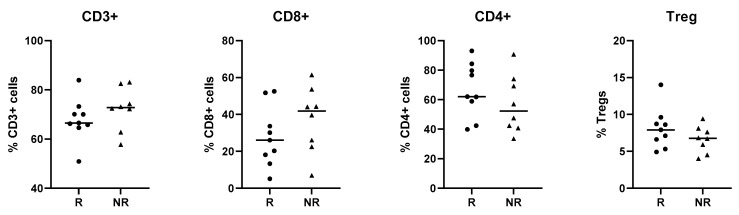
T cell proportions in NET patients. The overall T cell percentages were assessed for all NET patient samples that had been sorted for NanoString analysis. The % CD3+ cells (**left** graph) is derived from the ‘cells’ population in the gating strategy. CD8+ and CD4+ cells are both derived from the CD3+ population (**middle** graphs). The Treg population is derived from the CD4+ cells (**right** graph).

Following sorting for the T cell sub-populations, the percentage of each population was assessed. The overall CD3+ T cell populations were similar for both responders and non-responders. Although the proportion of CD8+ Tc cells tended to be higher in non-responders, while the CD4+ Th cells tended to be lower in non-responders (Appendix A Figure A2), these differences were not statistically significant. The patient, treatment, and interaction analysis, as outlined in the methods section, resulted in many genes that significantly (*p* ≤ 0.05) changed, and they are listed in the Supplementary Excel File S1. N = 26, 80, and 72 for patient, treatment, and interaction analysis, respectively for Tc cells; N = 33, 39, and 19 for Th, and N = 20, 36, and 15 for Treg cells. Table 2 summarizes the top 20 genes (i.e., lowest *p* values) for each of the analyses performed. The volcano plots in Figure 2 show the statistical significance (*p* value) versus the magnitude of change (fold change) for each of the nine categories in Tc, Th, and Treg).

The top 20 genes (lowest *p*-value) for each of the analyses performed are shown; red represents negative *t* values, and green represents positive *t* values. For patient effect analysis, positive and negative *t*-values are associated with higher or lower gene expression levels, respectively, in responders relative to non-responders. For treatment effect, positive and negative *t*-values are associated with higher or lower gene expression levels, respectively, after treatment relative to pre-treatment (regardless of outcome). For the interaction effect, positive and negative *t*-values are associated with ‘greater’ or ‘lesser’ effects, respectively, observed in responders relative to non-responders.

#### 3.2.3. Treatment Response Effect

**Figure 3:**
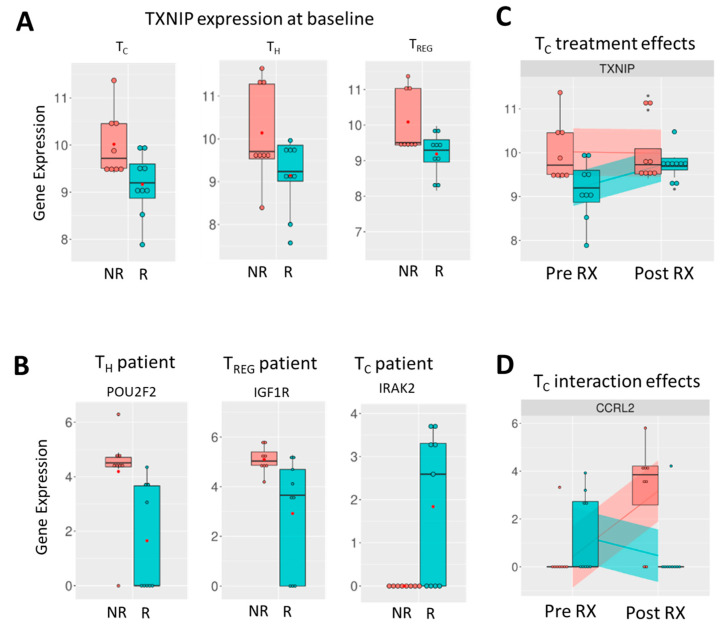
Expression of genes of note from transcriptional analysis of in vivo lanreotide treatment in T cell subsets from NET patients. (**A**) Expression of TXNIP in non-responders (red bar) and responders (teal bar) in Tc (**left** graph), Th (**middle** graph), and Treg cells (**right** graph). (**B**) Most significantly altered genes between non-responders and responders pre-treatment shown for Th (**left** graph), Treg (**middle** graph), and Tc cells (**right** graph). (**C**) Expression of TXNIP in Tc cells pre- and post-treatment in non-responders and responders indicates upregulation with lanreotide treatment in responders only. (**D**) Interaction effect, i.e., significant change in gene expression between non-responders and responders following treatment shown for CCRL2 in TC cells (**right** graph).

The correlation with treatment response (Table 2) indicates that genes that were most significantly different between responders and non-responders pre-treatment are observed in the immune modulatory Th cells and Treg cells. These genes are predominantly lower (highlighted red in Table 2) in responders, with 17 out of the top 20 genes in Th and Tregs having a negative *t* value. In Tc cells, there is an equal balance between upregulated (10/20) and downregulated (10/20) genes. Of note, one gene found with lower expression in responders in all T cell subpopulations is TXNIP, the gene encoding thioredoxin-interacting protein. (Figure 3A) In Th cells, the expression of POU2F2, the gene that encodes Oct-2, is significantly lower in responders (*t* value −3.098, *p* value 0.002). (Figure 3B, left graph) In Treg cells, IGF1R, which encodes IGF1 receptor protein, is the most significantly differentially expressed gene and is higher in non-responders (*t* value −3.014, *p* value 0.003). (Figure 3B, middle graph) In Tc cells, the most significantly differentially expressed gene is IRAK2, showing higher expression in some responders (*t* value 3.290, *p* value 0.001) (Figure 3B, right graph).

#### 3.2.4. Treatment Effect

In contrast to the in vitro data obtained from healthy donor T cells, where no significant effects of lanreotide exposure were observed for the parameters studied, the in vivo data demonstrated significant treatment-induced effects (Figure 2). These treatment effect correlations are also evident from Table 2 and show that the majority of the top 20 affected genes in all three T cell subpopulations are higher following treatment (highlighted green).

Genes identified as significantly changed (*p* ≤ 0.05) in the treatment effect analysis included several members of the NF-kB, Janus kinase/signal transducer and activator of transcription protein (Jak/STAT), and MAPK (NFKBIA, TRAF6, TFEB, CARD11, PSMD7, MAP2K4, FOXJ1, IRF5) signaling pathways.

#### 3.2.5. Interaction Effect

Lanreotide treatment had less effect on the immune-related gene expression profiles in effector Tc cells of responders evident from the predominantly (18/20) negative *t*-values in the top 20 affected genes in the interaction effect analysis (Table 2). For the immune modulatory Th and Treg cells, the interaction effects were more balanced, with 11/20 negative *t* values observed for each. PDCD1, the gene encoding the immune checkpoint protein PD-1 (CD279), is significantly changed following treatment and upregulated in the non-responders following treatment in only the Treg cells. Of note, the aforementioned TXNIP gene, which was found with lower baseline expression in responders in all T cell subpopulations (Figure 3A), was significantly changed only in the effector Tc cells of responders where upregulation following treatment is observed (*t* value 3.190, *p* value 0.001) (Figure 3C). CCRL2 (C-C motif chemokine receptor-like 2) was the most significantly altered gene in Tc cells following lanreotide treatment (*t* value −4.257, *p* value 0.000).

To determine if an association could be found between the changes in the individual genes analyzed and specific pathways, a GSEA pathway enrichment analysis using gProfiler2 was performed by probing the Kyoto Encyclopedia of Genes and Genomes (KEGG) and Reactome databases. The analysis results are summarized in Table 3, with most pathways being identified from the Reactome database. Pathways identified include those involved in stress response and infection. Notable pathways are described in more detail in the discussion section.

## 4. Discussion

This study demonstrates that despite the presence of SSTR receptors on T cells, there is no effect in vitro of lanreotide treatment on the functional and survival parameters tested of healthy donor Tc, Th, and Treg cells. In contrast, the analysis of the same T cell subsets after in vivo lanreotide treatment of NET patients revealed differences in immune response-associated genes and correlations with clinical response, which raises important implications on the therapeutic landscape of patients with NET.

Lanreotide mechanistically binds to SSTR receptors; however, there is differential affinity for specific receptors. In the context of studying lanreotide effects on immune cells, we first confirmed SSTR expression on T cells and our findings support previously published data that establish the presence of SSTRs on T cells [17,25]. It should be noted that reported differential SSTR expression levels may vary based on the detection method applied (including fluorescently-labeled ligands, RT-PCR, and antibody staining) and/or the maturation or activation state of the T cells [14,15,16,17,18,19]. In the present study, SSTRs were exclusively detected by antibody staining, and the three examined T cell subsets showed similar SSTR expression profiles, with predominantly high levels of SSTR2 but low or no expression of the other SSTRs. Our findings are similar to previous studies looking at the differential expression of SSTRs in T cells [17,18].

Next, we studied the effects of in vitro lanreotide exposure on human healthy donor immune cells.

The functional immune response parameters examined in healthy donors were unaffected by a range of lanreotide concentrations (0.01–10 mcM). This concentration range includes the clinically achievable level of 6.059 ng/mL (5.5 mcM) that is within goal mean trough serum lanreotide concentrations (5.3 to 8.6 ng/mL or 5.3 to 8.6 mcM) for the treatment of gastroenteropancreatic NET patients [26]. The absence of in vitro lanreotide-induced effects on T-cell survival (Figure 1B right plot) is functionally corroborated by the intracellular cytokine production assays (Figure 1B left and middle plots) as well as the signal transduction assays (Figure 1C). This finding is consistent with previous studies, which have shown that lanreotide has no cytotoxic effect when administered alone and, in clinical practice, is a cytostatic drug [27]. It should be noted that these in vitro studies were limited to studying the lanreotide effects on the NFAT, NF-kB, and ERK1/2 pathways. The focus on these pathways was based on their key roles in T cell activation [28] and prior reports on the modulatory effects of lanreotide on ERK signaling [29]. As evident from the much broader NanoString analysis performed to study in vivo treatment effects, in vitro lanreotide effects on other signaling pathways cannot be ruled out.

To examine the in vivo effects of lanreotide on the immune cells of NET patients, we obtained peripheral blood mononuclear cells from 17 patients before and after treatment and retrospectively studied them for gene and protein expression profiles in sorted T cell subsets (Tc, Th, Treg) using a NanoString immune cell focused panel. There were no statistically significant proportional differences observed between the Tc, Th, and Treg subpopulations when comparing the responder group with the non-responder group; however, when analyzing the data for responders versus non-responders as well as treatment and interaction effects, differences were observed that could not have been predicted based on the in vitro data.

In analysis of responders versus non-responders, the TXNIP gene, which encodes for thioredoxin-interacting protein, showed lower expression in all T cell subpopulations in the responder group. This protein has been proposed as a tumor suppressor, and it will be interesting to explore its role in NET pathogenesis [30]. Additionally, POU2F2 showed significantly lower expression in Th cells of responders. POU2F2 encodes Oct-2 which is essential in immune response and its over-expression has been implicated in several cancers, including glioblastomas, breast, and pancreatic cancers [31,32,33]. This differential expression of Oct-2 in responders vs. non-responders could be an interesting prognostic marker, given its role in other types of cancer. Among Treg cells, IGF1R showed significantly higher expression in non-responders. This gene encodes the IGF1 receptor, a pro-survival, anti-apoptotic tyrosine kinase that has been shown to be over-expressed in pulmonary NETs [34]. In Tc cells, the IRAK2 gene showed higher expression in some responders. This gene encodes a protein of the same name that is implicated in interleukin-1 (IL-1) receptor-mediated mitogen-activated protein kinase (MAPK) and NF-kB signaling pathways [35].

In our interaction effects analysis, CCRL2 (C-C motif chemokine receptor-like 2), an atypical chemokine receptor expressed on macrophages, monocytes, and neutrophils, was the most significantly altered gene in Tc cells following lanreotide treatment (*t* value −4.257, *p* value 0.000). CCRL2 on macrophages has been shown to upregulate Tc cells in mouse models [36], suggesting that macrophages, or more likely, monocytes in peripheral blood were co-isolated during the sorting procedure of the T cell subpopulations.

It should be noted that for this analysis, no functional markers (activation or exhaustion markers) were included. Before treatment, we observed differential expression of genes between non-responders and responders consistent with a more functional acuity of the immune response in the latter. The observed gene expression profile is consistent with that of a chronic infection response, which may be limiting the function of Th cells through exhaustion. Studies have shown that cancer can act like a chronic infection and may be limiting Th function in non-responders [37]. This finding could be of predictive value to identify patients who would benefit from lanreotide treatment. Early trials used radionuclide scan positivity as part of the eligibility criteria to enroll patients for SSA treatment (CLARINET) [10,11,12]. Others (PROMID) did not, and patients still benefit from SSA, demonstrating that a positive scan radionuclide scan is not a predictor of response or benefit [38]. In current clinical practice, patients with NETs receive lanreotide initially without testing for accurate predictive biomarkers of its efficacy.

After treatment with lanreotide, TCR, Ras, PI3K-Akt, and general signaling pathways were upregulated in both responders and non-responders, with the most significant effect in CD8+ Tc cells. These pathways are responsible for the regulation, activation, and differentiation of T cells. TCR-driven intracellular signaling cascades initiate and regulate many aspects of T cell-mediated immune responses, and the intensity of TCR signaling leads T cells toward diverse effector lineages [39]. T-cell immune infiltrates are surrogates for effective immunological response and have been reported to be associated with improved recurrence-free survival in patients with intermediate-grade NETs [40].

When looking at pathway differences between responders and non-responders, the most enriched pathway was TGFb signaling in Tc cells. A number of positively enriched pathways were also seen in Treg cells in responders, and all were transcriptional signaling pathways involved in metabolism, proliferation, differentiation, and survival.

Taken together, our findings indicate that lanreotide has an inhibitory effect on T cell proliferation and activation. This is likely to attenuate the immune response even further in people who already have a suboptimal immune response to the tumor. On the other hand, those with a significant immune response at baseline (which were the clinical responders here) continue to benefit from therapy, probably because the immune response suppression generated by lanreotide is not complete.

The benefit can also be explained by the preservation of lanreotide’s direct and indirect anti-proliferative effect on the tumor which has been demonstrated repeatedly with multiple different mechanisms based on the type of receptor stimulated [41,42,43,44,45]. Somatostatin’s direct anti-proliferative effect is mediated via activation of SHP tyrosine phosphatases and ERK pathways [46,47,48,49]. Somatostatin induces apoptosis in cell lines examined via SSTR2 and SSTR3 [50,51]. Indirect effects include the suppression of growth hormone and growth factor release, which stimulate the proliferation of many cell types. Overall, our results concur with previous findings that suggest somatostatin may have anti-proliferative effects on lymphocytes [52,53,54].

Considering these findings, we hypothesize that combining lanreotide with medications that enhance the tumor microenvironment and immune response may help mitigate lanreotide’s negative effect on T cells by preserving tumor antigen presentation and activation of T cells, thereby improving response to lanreotide. We recognize that the small sample size limits the ability to draw firm conclusions. There have been studies examining the combination of lanreotide and other T cell-enhancing agents. For example, lanreotide has been explored in conjunction with IL-2 in the treatment of medullary thyroid carcinoma (MTC), with early evidence suggesting that it may have a role in the treatment of advanced MTC [55]. These findings are awaiting confirmation in larger randomized studies. Also, preliminary results of combination immunotherapy, pembrolizumab, with lanreotide show promising activity in non-resectable, recurrent, or metastatic well or moderately differentiated NETs after progression on SSAs [56].

Certain antigen-presenting cell-specific genes were found to have been significantly changed in the T cell populations. These include B cell genes (CD79B, MS4A1, TNFSF13), NK cell genes (KLRC2, KIR3DL2), and myeloid/macrophage/dendritic genes (ITGB2, ITGA4, CLEC4A, LAMP3). The present analysis cannot provide evidence of whether these populations may also include circulating monocytes differentiated into CD3+TCRαβ+ and CD3+TCRαβ− macrophages or tightly bound T cell: monocyte conjugates. There are published reports of monocyte gene expression being unexpectedly detected in human CD4+ memory T cells. It has been confirmed that T cell-monocyte complexes exist in vivo in peripheral blood rather than being formed ex vivo because of sample manipulation [57]. The interaction of T cells with antigen-presenting cell (APC) immune system components demonstrates the relevance of additional characteristics in the tumor microenvironment that were not accounted for in the in vitro experimental conditions.

Collectively, our results suggest significant lanreotide pharmacodynamic effects on immune function that correlate with response in NET patients. The in vivo immune effects of lanreotide seen in the absence of in vitro effects may reflect the relevance of environmental parameters such as the interactions with APC components of the immune system not accounted for under the experimental in vitro conditions. Alternatively, it could reflect the difference between normal T cells, which were used for the in vitro studies, versus T cells from NET patients, which were used for the in vivo studies, or the more limited focus on the NFkB, NFAT, and ERK signaling pathways in the in vitro studies. Even for these three pathways, lanreotide treatment effects were observed in vivo but not in vitro. A limitation of the current study is that the effects of in vitro lanreotide exposure on NET patient T cells were not investigated. Additionally, the SSTR profiles observed in our study differ from some previous studies, which need further exploration. Microenvironment-related influences as well as differences introduced through the detection methods used in our study on in vitro versus in vivo analyses need more insight. There is a lack of reliable animal models or human systems to explore the downstream effects of lanreotide on an intact immune system; this underscores the importance of developing better models to assess immune effects in NETs. The small sample size of our study is a drawback that may limit the generalizability of our findings as well as limit the scope for evaluating potential confounders. Given the long survival in NET patients, PFS was used as a surrogate of efficacy compared to OS, which would need longer follow-up beyond the scope of the study. We did not include a control group not receiving lanreotide in the in vivo analysis owing to our study design, which explored response to lanreotide treatment: ‘non-responders’ vs. ‘responders’. Additionally, due to limited funding and early closure, which resulted in less recruitment of subjects, we could not validate our reported gene expression patterns as predictive biomarkers for treatment response in a larger and more diverse population. This would be the next step in trying to replicate our findings in a future study. We also encourage others to replicate the study at their centers to validate our findings in larger and more diverse study populations and explore more cytokine pathways.

The primary focus of the present work was to determine the treatment effects of lanreotide on T cell subsets. The data presented herein suggest that the in vivo effect of lanreotide on T cells is a general effect and indiscriminate between responders and non-responders. However, the consequences of these changes may be different depending on ‘baseline’ immune response-associated parameters consistent with chronic infection. It should be noted that, alternatively, the therapeutic benefits of lanreotide on NET patients are altogether independent of its effects on the immune system and that clinical response is related to the direct effects on the malignant cells or is a combination of both.

Given the observed in vivo effects of lanreotide on T cells from NET patients, it is possible that immunological profiling of patients with advanced NETs could identify potential biomarkers for response to lanreotide treatment.

## 5. Conclusions

Our study sheds light on the immune effects of lanreotide as a potential mechanism that underlies its therapeutic efficacy in neuroendocrine tumors. We demonstrated and characterized somatostatin receptors in healthy T cells but observed no differences before and after lanreotide treatment on cytokine production or T cell apoptosis in healthy donor T cells. However, in samples taken from neuroendocrine tumor patients, we saw significant differential expression of genes involved in cytokine signaling, upregulation of ubiquitination, and proteasome degradation, which correlated with clinical response. This creates exciting avenues to explore novel treatment modalities affecting the immune pathways in gastroenteropancreatic neuroendocrine tumors.

## Figures and Tables

**Table 1:** Patient Demographics.

	Responders (N = 9)	Non-Responders (N = 8)
**Age Median (range), y**	69 (34–79)	66 (50–81)
**Origin**		
**pNET**	5	4
**Intestinal NET**	4	3
**Unknown**	0	1
**Metastatic sites, N**		
**Liver**	6	6
**Nodes**	1	2
**Lung**	0	1
**Skin**	0	1
**Peritoneum**	1	0
**Ki 67, N**		
**Not specified**	1	2
**<3%**	5	0
**3–20%**	3	4
**>20%**	0	2
**Differentiation, N**		
**Not specified**	1	0
**Well-differentiated**	8	7
**Moderately differentiated**	0	1

**Table 2:** The patient-, treatment-, and interaction-analysis of lanreotide exposure in vivo in 3 T cell subsets (Tc, Th, Treg).

Patient Effect			Treatment Effect			Interaction Effect		
Tc	Th	Treg	Tc	Th	Treg	Tc	Th	Treg
IRAK2	POU2F2	IGF1R	CCRL2	LAG3	MAP2K1	CCRL2	IL5RA	HLA_DRB3
TXNIP	TNFRSF14	ECSIT	BCL2L1	MS4A1	PDCD1	TNFRSF13C	IRAK2	IRF5
IL6	NUP107	CCND3	FCGR2A	TFEB	KIR_Inhibiting _Subgroup_1	HLA_DRB4	IFI16	FCER1G
TRAF6	ATG5	ITGA4	MRC1	CASP3	EP300	IRAK2	NUP107	MFGE8
VEGFC	TFEB	C1QBP	TLR6	EGR1	TP53	FEZ1	C1QBP	ATF2
SPA17	IL21R	RELA	KLRC2	GTF3C1	NFKBIA	TXNIP	LAG3	HLA_DPB1
KLRC2	CREB1	MERTK	CLEC4C	BCL10	FCER1G	CMKLR1	BID	LAMP3
SIGIRR	AKT3	CD7	HLA_DRB4	PSMD7	CD80	CDKN1A	BCL10	KIR_Inhibiting _Subgroup_1
TNFSF12	IL17RA	CREB1	CD79B	TRAF6	TYK2	CLEC4A	PSMD7	PDCD1
TNFRSF13C	TXNIP	TXNIP	MASP1	XCL2	HLA_DPB1	MICA	MS4A1	IGF1R
CREB1	GZMK	LAMP3	NFKBIA	DDX58	CCR2	TMEFF2	TXNIP	TNFRSF10C
MAP2K4	BCL2	PIN1	TLR8	NFKBIA	NUP107	CD70	CCL4	RELA
CD3E	CASP3	CXCR5	TNFRSF13C	CXCR6	SH2D1A	FAS	TRAF6	EP300
MIF	MAP2K1	ITGAL	DMBT1	CD3EAP	TFRC	TNFRSF12A	CXCR3	TXNIP
FOS	HLA_DPB1	BCL2	LILRB1	CR1	CCR9	TRAF6	JAM3	IL13
CARD11	ATM	SMAD3	YTHDF2	IRAK2	IL8	FOXJ1	IL18RAP	ECSIT
ITGB2	IRAK4	CARD11	IL6ST	IL5RA	TLR10	TNFSF13	NOTCH1	IL1R1
IL11	CD40LG	TCF7	FUT7	CXCL5	IRF5	IFIT1	PTGDR2	SPACA3
SMAD3	ITGAL	RUNX1	MAP2K4	EOMES	MFGE8	IFIH1	TNFRSF14	XCR1
FAS	PSMB9	IRAK4	KIR3DL2	S100A8	IL6ST	LILRB1	GTF3C1	BCL10

**Table 3:** Gene Set Enrichment Analysis (GSEA) was performed by probing the Kyoto Encyclopedia of Genes and Genomes (KEGG) and Reactome databases.

	Pathway	NES	*p* Adjusted
**Patient Effect**			
Th	NOD-like receptor signaling pathway	−1.527	0.0469
	Yersinia infection	−1.594	0.0369
	Herpes simplex virus 1 infection	−1.624	0.0208
	Human immunodeficiency virus 1 infection	−1.628	0.0234
	Regulation of actin cytoskeleton	−1.780	0.0208
Tc	-		
Treg	-		
**Treatment Effect**			
Th	Cell surface interactions at the vascular wall	1.705	0.0354
	Disease	−1.386	0.0354
	RNA Polymerase II Transcription	−1.580	0.0354
	Generic Transcription Pathway	−1.610	0.0326
	Gene expression (Transcription)	−1.652	0.0203
	Cellular responses to stress	−1.780	0.0203
	Cellular responses to stimuli	−1.780	0.0203
	Deubiquitination	−1.844	0.0345
	Post-translational protein modification	−1.852	0.0184
	Purinergic signaling in leishmaniasis infection	−1.867	0.0394
	Cell recruitment (pro-inflammatory response)	−1.867	0.0394
	Metabolism of RNA	−1.892	0.0203
	Metabolism of proteins	−1.902	0.0054
	Ub-specific processing proteases	−1.908	0.0203
Tc	Metabolism of proteins	−1.765	0.0485
	cGMP-PKG signaling pathway	1.833	0.0234
	Ras signaling pathway	1.817	0.0234
	Focal adhesion	1.746	0.0432
	Rap1 signaling pathway	1.716	0.0493
	Cell adhesion molecules	1.678	0.0432
	Yersinia infection	1.663	0.0432
	T cell receptor signaling pathway	1.662	0.0432
	PI3K-Akt signaling pathway	1.632	0.0432
Treg	-		
**Interaction Effect**			
Th	-		
Tc	Signaling by TGF-beta Receptor Complex	2.003	0.0438
	Signaling by TGFB family members	2.003	0.0438
Treg	Host Interactions of HIV factors	1.693	0.0171
	HIV Infection	1.683	0.0171
	TCF dependent signaling in response to WNT	1.681	0.0171
	Cell surface interactions at the vascular wall	1.651	0.0171
	TCR signaling	1.638	0.0276
	Signaling by ROBO receptors	1.622	0.0350
	Signaling by WNT	1.608	0.0350
	Transcriptional regulation by RUNX3	1.581	0.0350
	Diseases of signal transduction by growth factor receptors and second messengers	1.548	0.0350
	Generic Transcription Pathway	1.503	0.0276
	RNA Polymerase II Transcription	1.469	0.0350
	Gene expression (Transcription)	1.459	0.0469

Encyclopedia of Genes and Genomes (KEGG) and Reactome databases; NES: normalized enrichment score.

## Data Availability

The data presented in this study are available on request from the corresponding author due to (human subjects privacy and protected health information).

## Supplementary Materials

The following supporting information can be downloaded at: https://www.mdpi.com/article/10.3390/cancers16173104/s1. File S1: The patient-, treatment-, and interaction-analysis of nanostring data: listing of genes that significantly (*p* ≤ 0.05) changed. The top 20 genes for each analysis are highlighted in yellow.

## Appendix A

**Table A1:** Antibodies used in the study.

Healthy Donor T Cell Phenotyping				
Antigen	Fluor	Clone	Vendor	Cat #
CD3	APCH7	SK7	BD Pharmingen	560176
CD4	PECy7	SK3	BD Pharmingen	557852
CD8	PerCP	SK1	BD Biosciences	347314
CD25	PE	2A3	BD Biosciences	341009
CD127	BV421	HIL-7R-M21	BD Horizon	562436
**SSTR expression**				
Rb anti-SSTR1	N/A	UMB7	Abcam	ab137083
Ms anti-SSTR2	N/A	402038	Biotechne	MAB4224
Rb anti-SSTR3	N/A	UMB5	Abcam	ab137026
Rb anti-SSTR4	N/A	Polyclonal	Abcam	ab28578
Ms anti-SSTR5	N/A	394401	Biotechne	MAB4448
Dky anti Rb IgG	AF647	N/A	Jackson ImmunoResearch	711-605-152
Gt anti Ms. IgG	AF647	N/A	Jackson ImmunoResearch	115-605-003
**Cytokine expression**				
IL-2	APC	5344.111	BD FastImmune	341116
IFN-γ	BV510	B27	BD Horizon	563287
**Transcription Factor signaling**				
NFAT1	N/A	D43B1	Cell Signaling	5861
NF-κB p65	N/A	D14E12	Cell Signaling	8242
ERK1/2	N/A	137F5	Cell Signaling	4695
**NanoString antibodies**				
CD3	FITC	UCHT1	Biolegend	300406
CD8	PerCPCy5.5	HIT8a	Biolegend	300924
CD4	BV510	A161A1	Biolegend	357420
CD25	PE	BC96	Biolegend	302606
CD127	PECy7	A019D5	Biolegend	351320
LiveDead	Violet	N/A	ThermoFisher	L23105

**Table A2:** In vitro effects of lanreotide exposure on the PMA/Ion- or anti-CD3/CD28-activation of NFAT1, NF-κB(p65) or ERK1/2. Mean similarity score (SS) values were obtained from 10 healthy donors with the associated standard errors (SE).

NFAT1	Lanreotide (µM)	NFAT1 Baseline Mean SS (SE)	NFAT1 PMA/Ion Mean SS (SE)	NFAT1 CD3/CD28 Mean SS (SE)
T-helper	0	0.06 (0.04)	2.54 (0.07)	0.42 (0.05)
	0.5	0.04 (0.04)	2.69 (0.05)	0.42 (0.05)
	5	0.09 (0.06)	2.66 (0.05)	0.40 (0.06)
T-cytotoxic	0	−0.04 (0.05)	2.58 (0.08)	0.36 (0.06)
	0.5	−0.06 (0.05)	2.68 (0.06)	0.33 (0.05)
	5	−0.03 (0.05)	2.68 (0.05)	0.34 (0.06)
Treg	0	0.26 (0.04)	2.52 (0.07)	0.60 (0.05)
	0.5	0.23 (0.05)	2.67 (0.05)	0.56 (0.06)
	5	0.25 (0.05)	2.60 (0.05)	0.56 (0.07)
**NF-κB/p65**	**Lanreotide** **(µM)**	**NF-κB/p65 Baseline** **Mean SS (SE)**	**NF-κB/p65 PMA/Ion** **Mean SS (SE)**	**NF-κB/p65 CD3/CD28** **Mean SS (SE)**
T-helper	0	0.25 (0.12)	2.04 (0.15)	1.04 (0.05)
	0.5	0.25 (0.11)	2.04 (0.13)	0.98 (0.07)
	5	0.16 (0.12)	2.07 (0.14)	1.05 (0.07)
T-cytotoxic	0	0.15 (0.12)	1.94 (0.12)	0.86 (0.07)
	0.5	0.14 (0.10)	1.92 (0.13)	0.83 (0.08)
	5	0.05 (0.12)	1.97 (0.13)	0.86 (0.09)
Treg	0	0.50 (0.14)	2.13 (0.13)	1.26 (0.07)
	0.5	0.53 (0.10)	2.09 (0.12)	1.18 (0.10)
	5	0.41 (0.14)	2.12 (0.14)	1.24 (0.09)
**ERK1/2**	**Lanreotide** **(µM)**	**ERK1/2 Baseline** **Mean SS (SE)**	**ERK1/2 PMA/Ion** **Mean SS (SE)**	**ERK1/2 CD3/CD28** **Mean SS (SE)**
T-helper	0	1.12 (0.04)	1.44 (0.07)	1.17 (0.03)
	0.5	1.10 (0.05)	1.51 (0.07)	1.22 (0.03)
	5	1.15 (0.04)	1.47 (0.06)	1.20 (0.03)
T-cytotoxic	0	1.02 (0.05)	1.39 (0.09)	1.10 (0.07)
	0.5	0.98 (0.05)	1.47 (0.08)	1.09 (0.05)
	5	1.05 (0.05)	1.42 (0.09)	1.06 (0.05)
Treg	0	1.33 (0.05)	1.53 (0.06)	1.37 (0.04)
	0.5	1.32 (0.05)	1.61 (0.07)	1.40 (0.04)
	5	1.38 (0.06)	1.60 (0.07)	1.37 (0.03)
